# Predicting Job Performance in the Nonprofit Sector: The Role of Self-Efficacy and Type D Personality Traits in Greek Non-governmental Organization Employees

**DOI:** 10.7759/cureus.100690

**Published:** 2026-01-03

**Authors:** Nikolina Kapsali, Chrysovalantis Karagkounis, Georgios Manomenidis, Thalia Bellali

**Affiliations:** 1 Psychology, Alzheimer Hellas, International Hellenic University, Thessaloniki, GRC; 2 Nursing, Faculty of Nursing, International Hellenic University, Thessaloniki, GRC; 3 Faculty of Health Sciences, Nursing Democritus University of Thrace, Alexandroupolis, GRC; 4 Nursing, International Hellenic University, Thessaloniki, GRC; 5 Health Sciences, European University Cyprus, Nicosia, CYP

**Keywords:** job performance, non-governmental organizations, occupational stress, self-efficacy(se), type d personality

## Abstract

Background: Job performance (JP) in non-governmental organizations (NGOs) is shaped by both facilitating and inhibiting psychological factors. While self-efficacy (SE) enhances motivation and task execution, Type D personality, characterized by negative affectivity (NA) and social inhibition (SI), has been associated with poor work outcomes. However, limited research has examined these variables in nonprofit environments. This study aimed to explore the predictive role of SE and Type D personality on in-role and extra-role job performance among NGO employees in Greece.

Objective: This study examines whether SE and Type D personality traits (NA and SI) predict in-role and extra-role job performance among employees working in Greek NGOs.

Methods: A cross-sectional study was conducted between January and March 2025 among 175 NGO employees using convenience sampling. Participants completed the Job Performance Scale (JPS), the Type D Personality Questionnaire (DS14), and the General Self-Efficacy Scale (GSE). Data were analyzed using non-parametric tests and multivariate linear regression.

Results: Multiple regression analyses revealed that SE consistently emerged as a positive predictor of JP, enhancing both in-role (β = 0.350, p < 0.01) and extra-role performance (β = 0.342, p < 0.01). In contrast, the Type D personality trait of SI was a negative predictor of extra-role (β = −0.158, p < 0.05) and overall JP (β = −0.450, p < 0.01), while NA was not significant. Being married was associated with lower in-role (β = −0.768, p < 0.05) and overall JP (β = −0.203, p < 0.01), and male gender predicted higher extra-role performance (β = 1.319, p < 0.05). The models explained 27.9% of the variance in in-role JP, 21.5% in extra-role JP, and 29.3% in overall JP.

Conclusions: SE and SI emerged as key psychological predictors of performance in the NGO workplace. Interventions that strengthen SE and reduce SI, such as cognitive-behavioral training, peer mentoring, and psychologically safe work climates, may enhance employee effectiveness. These findings are especially relevant for Greek NGOs operating under economic and organizational constraints. Future longitudinal and mixed-methods studies are recommended to explore causal pathways and inform workforce development strategies in mission-driven sectors.

## Introduction

In the last two decades, non-governmental organizations (NGOs) have gained international recognition for their vital contributions to society [1]. Their popularity derives from providing essential services to vulnerable groups, such as the homeless, elderly, and marginalized, while also actively advocating for policy changes and raising public awareness to address societal challenges [2]. Beyond these efforts, NGOs also engage in broad-scale initiatives, including human rights advocacy, conflict mediation, environmental activism, health promotion, and political analysis [2]. In addition, NGOs are uniquely positioned to build strong local connections and implement targeted interventions effectively, given their expertise and deep community ties [1].

NGOs constitute a diverse array of autonomous, nonprofit organizations that defy a singular, universally accepted definition [3]. Broadly, NGOs can be categorized according to their core objectives, such as humanitarian assistance, developmental programs, or the promotion of public awareness. Humanitarian NGOs typically address gaps in state welfare by offering social assistance to vulnerable groups, including homeless individuals, substance users, migrants, victims of human trafficking, and individuals with rare diseases [4]. In contrast, developmental NGOs concentrate on implementing programs in underdeveloped or developing regions to bring about transformative changes in daily life [4]. NGOs can also be categorized by their level of operation, whether local, regional, national, or international [5]. For instance, Alzheimer’s Associations globally work to raise awareness and provide services to dementia patients and their caregivers. In essence, NGOs are autonomous, nonprofit entities that function outside governmental control [6].

A fundamental determinant of NGO success and sustainability is the Job Performance (JP) of its employees. JP is defined as the combination of skills, knowledge, and motivation that align with organizational goals and is measurable by the employee’s contribution to these objectives [7]. Previous research conceptualizes JP as the overall value of an employee’s behaviors, both directly and indirectly, toward achieving organizational objectives [8,9]. JP can be categorized into two primary dimensions, in-role performance and extra-role performance [10]. In-role performance refers to the fulfillment of job responsibilities, encompassing tasks explicitly outlined in job descriptions [11]. Extra-role performance refers to discretionary behaviors that go beyond formal job duties, such as effective communication, proactive behavior, and maintaining personal discipline [10,12]. These behaviors, while not contractually required, are essential for organizational efficiency and adaptability. Importantly, employees must believe in their ability to complete tasks effectively to achieve high performance levels [13].

The psychological underpinnings of JP can be further understood through established theoretical models. For instance, Bandura’s Social Cognitive Theory [13,14] highlights self-efficacy (SE) as a central cognitive mechanism that influences motivation, persistence, and performance outcomes. Likewise, Campbell’s Job Performance Model [9,15] and the Job Demands-Resources (JD-R) model [16] emphasize the role of personal characteristics, such as personality and perceived competence, in shaping work behaviors. Within this framework, two psychological factors stand out and are discussed in greater detail below: Type D personality and SE.

The JP of employees is influenced by several factors, both positively and negatively. One notable determinant is Type D personality, characterized by negative affectivity (NA) and social inhibition (SI). NA reflects the tendency to experience persistent negative emotions, while SI involves the suppression of emotional expression due to fear of rejection or disapproval [17]. Individuals with Type D personality often report feelings of pessimism, anxiety, and irritation, and tend to avoid sharing negative feelings [18]. These traits can lead to workplace stress, exhaustion, cynicism, and reduced JP [19,20]. For example, a study in Poland found that nurses exhibiting Type D personality characteristics were more likely to use ineffective patient-care techniques, which negatively impacted their JP [21]. Likewise, Abraham [22] reported that individuals with Type D personality are more likely to give up when faced with challenges, change occupations, and experience a lower level of job satisfaction.

Another critical psychological factor influencing JP is SE, defined as an individual’s belief in their capability to perform tasks and achieve goals successfully [14]. Employees with high SE tend to exhibit greater job satisfaction, stronger commitment to their work, and reduced absenteeism [23,24]. SE is also associated with resilience, as employees with high SE are more likely to persist through workplace challenges [25]. In contrast, employees with low SE display avoidance of complex tasks and are more likely to give up when encountering difficulties [26-28]. Enhancing SE has been shown to positively influence the quality of care provided in various sectors, including healthcare [29].

Despite the well-established theoretical foundations linking psychological constructs such as SE and personality traits to JP, recent reviews underscore the lack of context-specific investigations in the nonprofit sector. For instance, Krijgsheld et al. [30] noted that most empirical studies on job performance have focused on clinical or corporate settings, with NGO and nonprofit environments remaining largely overlooked. Hameli et al. [31] similarly emphasized that while SE and Type D personality have been widely studied in healthcare, education, and military contexts, their relevance in emotionally demanding, resource-limited workplaces such as NGOs is insufficiently understood. This research gap is particularly critical given the unique psychosocial landscape of NGO work, characterized by high emotional labor, blurred role boundaries, and systemic constraints [32]. Moreover, few studies to date have explored the combined impact of psychological facilitators (e.g., SE) and inhibitors (e.g., SI) on different facets of JP, which may hinder the development of holistic and targeted organizational strategies [6,21].

Employees in NGOs often operate under unique and demanding conditions that distinguish them from their counterparts in the public or private sectors. They are frequently exposed to emotionally intense environments, resource constraints, job insecurity, and high workloads, all of which can contribute to elevated stress levels and emotional exhaustion [33]. Moreover, many NGO employees are driven by strong intrinsic motivation, values-based commitment, and a sense of mission [34], which may interact differently with psychological traits such as SE and personality dimensions. These distinctive features underscore the importance of examining how internal psychological resources, like SE, and vulnerabilities, such as Type D personality, affect JP within this specific population. The decision to focus on NGO employees was thus motivated by the need to better understand the interplay between psychological characteristics and performance in a sector where human capital is both highly committed and highly vulnerable.

Given the growing global significance of NGOs, there is increasing interest in identifying the psychological and behavioral factors that influence employee JP in these organizations. Despite extensive research on JP, SE, and Type D personality in other professional contexts, such relationships remain underexplored in the NGO sector, particularly within the Greek context. Accordingly, this study investigates whether Type D personality traits (NA and SI) and SE significantly predict in-role and extra-role JP among NGO employees in Greece. To the best of our knowledge, this is the first empirical study in Greece to investigate these psychological variables in relation to JP in nonprofit settings. By focusing on this specific workforce, the present research contributes to the existing literature and offers practical insights for enhancing employee well-being and organizational effectiveness in NGOs.

## Materials and methods

Study design and setting

A cross-sectional descriptive study was conducted in various NGOs in Greece using a convenience sampling method. The participating NGOs included: (1) Alzheimer Athens, (2) the Association for Regional Development and Mental Health (EPAPSY), (3) the Hellenic Society of Alzheimer's Disease & Related Disorders, Alzheimer Hellas, and (4) The Smile of the Child. Participants represented a diverse range of professional roles, including physicians across various specialties, nurses and nursing assistants, social workers, psychologists, physiotherapists, and administrative personnel.

Data collection took place from January 1 to March 30, 2025. The survey was distributed electronically via the official communication channels of the participating organizations. Specifically, the research team provided the survey link to the organizations, which then forwarded it to their employees through internal email lists. Data collection was conducted entirely online. A detailed study information sheet accompanied the questionnaire, outlining the purpose of the research, the voluntary nature of participation, and assurances regarding anonymity and confidentiality. Participants were informed that they could withdraw from the study at any time without providing any justification. Consent was implied upon completion of the questionnaire.

Sample and data collection

A convenience sample of 175 NGO personnel participated in the study. The survey was distributed electronically through the internal email lists of the participating organizations, and only completed responses were accessible to the research team. Therefore, the exact number of employees who received the survey is unknown, and a response rate could not be calculated. Only fully completed questionnaires were included in the analysis, with missing responses excluded listwise.

Participants in the present study were required to meet specific inclusion criteria. Eligible individuals had to be currently employed in an NGO operating within Greece and possess a minimum of six months of continuous work experience in the NGO sector, ensuring sufficient exposure to the organizational environment. Only adults aged 18 years or older were considered, and participants needed to demonstrate adequate proficiency in the Greek language, as all survey instruments were administered in Greek. Furthermore, participation was entirely voluntary, and written informed consent was obtained from all respondents before data collection.

Exclusion criteria were equally defined to ensure the relevance and reliability of the sample. Individuals employed in governmental, private-sector, or for-profit organizations were excluded from the study. In addition, volunteers, interns, and short-term contractors without formal employment status were not eligible to participate. Those with less than six months of experience working in NGOs were also excluded to ensure consistency in participants’ familiarity with NGO-specific working conditions. Finally, any questionnaires with incomplete or inconsistent responses were removed from the final analysis.

Data were collected using an online form created via a General Data Protection Regulation (GDPR)-compliant online survey tool. Ethical approval (Pr Nr.: 247/2025 AI) was obtained before the study by the Bioethics Committee of the Greek Association of Alzheimer’s Disease and Related Disorders (GAADRD), and all procedures adhered to the principles outlined in the Declaration of Helsinki. The study ensured compliance with data protection regulations, and no personal identifiers were collected to maintain the confidentiality of participants’ responses.

Research instruments

Data were collected using an anonymous battery of validated research tools, which included the following: (a) Demographic and Occupational Characteristics, (b) the Job Performance Scale (JPS), (c) the Type D Personality Questionnaire (DS14), and (d) the General Self-Efficacy Scale (GSE).

Demographic and Occupational Characteristics

This questionnaire was constructed for the study, which included the following questions: age, gender, marital status, job title, years of experience, and organizational role.

Job Performance Scale (JPS)

The JPS consists of six items designed to measure JP. Three items assess in-role performance, reflecting behaviors directly related to job responsibilities (e.g., “I was able to carry out my work efficiently”), and the remaining three assess extra-role performance, capturing discretionary behaviors beyond formal duties (e.g., “I willingly attend functions not required by the organization, but which contribute to its overall image”). Responses are rated on a seven-point Likert scale, ranging from 0 (“not at all”) to 6 (“completely”). Higher scores indicate better performance, with possible score ranges of 0-18 for in-role performance, 0-18 for extra-role performance, and 0-36 for the total JPS score. The scale was originally developed by Goodman and Svyantek [35]. Cronbach’s alpha for the Greek version was 0.85 for in-role performance and 0.75 for extra-role performance [36].

Type D Personality Questionnaire (DS14)

The Type D Personality Questionnaire (DS14), developed by Denollet [37], assesses Type D personality traits through 14 items measuring two dimensions: NA and SI. NA captures the tendency to experience negative emotions (e.g., “I often find myself worrying about something”), while SI reflects the tendency to suppress emotions and avoid social interactions (e.g., “I find it hard to start a conversation”). Each dimension comprises seven items, scored on a five-point Likert scale ranging from 0 (“false”) to 4 (“true”). Total scores for each dimension range from 0 to 28, with higher scores indicating greater levels of NA and SI. The DS14 was previously translated and psychometrically evaluated in a Greek clinical population [38], demonstrating a stable two-factor structure and acceptable internal consistency across NA and SI subscales, with Cronbach’s alpha values of 0.88 for Negative Affectivity and 0.86 for SI.

General Self-Efficacy Scale (GSE)

The GSE, developed by Schwarzer and Jerusalem [39], measures individuals’ confidence in their ability to manage challenging tasks and unexpected situations successfully. The scale consists of 10 items (e.g., “I can always manage to solve difficult problems if I try hard”), scored on a four-point Likert scale ranging from 1 (“not at all true”) to 4 (“exactly true”). Higher scores indicate greater self-efficacy, with total scores ranging from 10 to 40. The GSE was translated and validated for use in Greece, with a Cronbach’s alpha of 0.78, ensuring its reliability within this population [40].

Use and Copyright Status of Measurement Tools

The Job Performance Scale (JPS), the Type D Personality Questionnaire (DS14), and the General Self-Efficacy Scale (GSE) are all previously published and validated instruments. The JPS is a copyrighted measure developed by Goodman and Svyantek [35] and was used in this study solely for non-commercial academic purposes, with full citation of the original source; no modifications were made to the scale. The DS14 [37] is an open-access instrument that is freely available for research use without the need for written permission, as stated by the original author. The GSE [39] is also an openly accessible tool for non-commercial academic research, according to the developers’ guidelines. All three instruments are properly cited in the manuscript. Since the JPS is a copyrighted tool, permission for its academic use was obtained.

Sample Size Calculation

An a priori sample size calculation was performed using G*Power 3.1 (Heinrich-Heine-Universität Düsseldorf, Düsseldorf, Germany). For a multiple linear regression model with five predictors, and based on prior literature and conventional benchmarks, an effect size of f² = 0.15 was selected according to Cohen’s guidelines for medium effects [41]. This choice is consistent with previous research examining psychological predictors of job performance, which commonly report medium-sized effects for self-efficacy and personality traits. Assuming a significance level of α = 0.05 and a statistical power of 0.80, the minimum required sample size was N = 92. The final study sample (N = 175) exceeded this requirement and was therefore considered adequate for the planned analyses.

Statistical analysis

Data were analyzed using IBM SPSS Statistics for Windows, Version 26 (Released 2018; IBM Corp., Armonk, New York). The distribution of numerical variables was tested for normality using the Kolmogorov-Smirnov test. Categorical variables are reported as frequencies and percentages, while continuous variables are presented as means with standard deviations. As the normality assumption was not met for all continuous variables, non-parametric statistical techniques were applied. The Mann-Whitney U test and the Kruskal-Wallis H test were used to assess differences in JP across categories such as gender, marital status, and educational attainment. Spearman’s rank-order correlation was used to explore associations between JP and other continuous or ordinal independent variables, while Pearson’s correlation was additionally conducted for continuous variables when the assumptions for parametric testing were met. Variables that demonstrated statistically significant relationships with JP in the bivariate analyses, using a significance threshold of p < 0.05, were subsequently included in a multiple regression model, with JP serving as the dependent variable.

## Results

A total of 175 questionnaires were received. Regarding gender distribution, males comprised 40.6% of the sample, while females comprised 59.4%. Participants not living with a partner or spouse accounted for 52.6% of the sample, whereas 47.4% lived with a partner or spouse. Concerning educational attainment, 9.7% had secondary education, 46.3% had tertiary education, and 44.0% held postgraduate qualifications (MSc/PhD). The mean age of NGO employees was 35.8 years (SD = 6.9). In terms of professional roles, psychologists constituted 56.6% of the sample, physicians 13.3%, nurses and nursing assistants 15.6%, and other professions 14.5%. Participants reported an average total working experience of 10.1 years (SD = 6.8) and an average of 6.0 years (SD = 4.9) in their current organization. Table 1 summarizes the demographic and occupational characteristics of the sample.

**Table 1:** Demographic and occupational characteristics of the study sample (N = 175). ^a^Mean (SD).

Characteristics	n (%)
Gender	
Male	71 (40.6%)
Female	59.4%
Age^a^	35.8 (6.9)
Marital status	
Without a partner/spouse	52.6%
With partner/spouse	47.4%
Educational Level	
Secondary education	9.7%
Tertiary education	46.3%
Master/PhD	44%
Profession	
Physicians	13.3%
Nurses/nursing assistants	15.6%
Psychologists	56.6%
Other	14.5%
Work experience	10.1 (6.8)
Work experience in NGO^a^	6.0 (4.9)

This table presents the distribution of participants across demographic variables (age, gender, marital status, and education) and occupational variables (profession, total work experience, and NGO work experience). Categorical variables are presented as frequencies and percentages, while continuous variables are shown as means with standard deviations.

Descriptive statistics for the scales and subscales (Type D personality, NA, SI, SE, in-role performance, extra-role performance, overall JP) are presented in Table 2. NA and SI had mean scores of 6.68 (SD = 4.77) and 7.35 (SD = 4.85), respectively, indicating moderate levels. SE had a relatively high mean score of 29.2 (SD = 3.15), suggesting that participants generally perceived themselves as capable in their roles. Regarding JP, the mean scores for in-role performance, extra-role performance, and overall JP were 13.58 (SD = 2.48), 12.99 (SD = 3.08), and 26.57 (SD = 4.97), respectively. These findings suggest that participants generally demonstrated strong performance in fulfilling core job responsibilities, while their engagement in discretionary, extra-role activities was moderate. The overall JP score indicates a satisfactory level of performance across both domains. Notably, the observed variability, particularly in extra-role and overall performance, may reflect differences in individual motivation, role expectations, or organizational support among participants.

**Table 2:** Descriptive statistics of psychological scales and job performance subscales.

Scales and Subscales	Means	SD
Type D personality		
Negative affectivity (NA)	6.68	4.77
Social inhibition (SI)	7.35	4.85
Self-efficacy	29.2	3.15
Job performance	26.57	3.95
In-role performance	13.58	2.48
Extra-role performance	12.99	3.08
Job performance (overall)	26.57	4.97

This table displays mean values and standard deviations for NA, SI, SE, and the three dimensions of job performance (in-role, extra-role, and overall). Higher values on each scale represent higher levels of the respective psychological or performance construct.

Bivariate analysis between independent variables and JP is displayed in Table 3. There was a strong positive correlation between SE and in-role performance (r = 0.46, p < 0.001), extra-role performance (r = 0.36, p < 0.001), and overall JP (r = 0.46, p < 0.001). More specifically, NA and SI were negatively correlated with all JP dimensions. NA was associated with lower in-role performance (r = -0.15, p < 0.05) and overall JP (r = -0.16, p < 0.05), while SI was linked to reduced in-role performance (r = -0.22, p < 0.01), extra-role performance (r = -0.22, p < 0.01), and overall JP (r = -0.24, p < 0.01). Moreover, there was a negative correlation between extra-role performance and age (r = -0.15, p < 0.05) and work experience (r = -0.17, p < 0.05). In contrast, marital status showed significant differences in both in-role and overall JP, with participants without a partner/spouse reporting higher scores. The Mann-Whitney U test indicated significant differences for in-role JP (U = 4459, p = 0.036) and overall JP (U = 4625, p = 0.043).

**Table 3:** Summary of bivariate analyses between independent variables and job performance (JP). Pearson’s r correlations were reported for continuous variables that met the assumptions for parametric testing, whereas Spearman’s rank correlations were used for ordinal or non-normally distributed variables. Non-parametric group comparisons (Kruskal–Wallis H and Mann–Whitney U) are also presented for categorical independent variables, as these tests assess significant differences rather than correlations. Significance levels: *p < 0.05, ** p < 0.01, *** p < 0.001. For Kruskal–Wallis H and Mann–Whitney U tests, p-values indicate significant differences between groups.

Independent Variable	Test Used	In-Role Performance	Extra-Role Performance	Job Performance (Overall)
Negative affectivity (NA)	Pearson’s r	-0.15*	-0.13	-0.16*
Social inhibition (SI)	Pearson’s r	-0.22**	-0.22**	-0.24**
Self-efficacy	Pearson’s r	0.46***	0.36***	0.46***
Age	Pearson’s r	-0.05	-0.15*	-0.12
Working experience	Pearson’s r	-0.04	-0.17*	-0.13
Working experience in NGO	Pearson’s r	0.00	-0.10	-0.06
Profession	Kruskal-Wallis H	p = 0.208	p = 0.611	p = 0.433
Educational level	Kruskal-Wallis H	p = 0.251	p = 0.609	p = 0.354
Gender	Mann-Whitney U	p = 0.774	p = 0.013	p = 0.094
Marital status	Mann-Whitney U	p = 0.036	p = 0.115	p = 0.043

This table presents the bivariate associations between all independent variables (NA, SI, SE, age, work experience, profession, educational level, gender, marital status) and the three dimensions of JP (in-role, extra-role, overall). Pearson’s correlation coefficients were used for continuous variables that met the assumptions for parametric testing, whereas Spearman’s rank correlation was applied to ordinal variables and to continuous variables that did not meet the normality assumption. Mann-Whitney U and Kruskal-Wallis H tests were used for categorical predictors. Significant associations are marked with *p < 0.05, **p < 0.01, ***p < 0.001. Positive coefficients indicate higher job performance with increasing values of the independent variable, whereas negative coefficients indicate the opposite.

Multivariate linear regression analysis results are presented in Tables 4, 5, and 6, respectively. According to the multiple regression analysis (Table 4), marital status, SE, and SI emerged as significant predictors of in-role performance. Specifically, being married was associated with a decrease in in-role performance (β = -0.768, p < 0.05), whereas higher self-efficacy was associated with improved in-role performance (β = 0.350, p < 0.01). This result differs from the bivariate findings in Table 3, in which unmarried participants showed higher in-role performance scores. This discrepancy arises because the regression model adjusts for additional variables simultaneously, revealing the unique contribution of each predictor. The overall model was statistically significant (F = 11.245, p < 0.001, R² = 0.279, adjusted R² = 0.261), explaining 27.9% of the variance in in-role job performance.

According to the multiple regression analysis presented in Table 5, gender, SE, and SI were significant predictors of extra-role performance. Men demonstrated higher extra-role performance (β = 1.319, p < 0.05), while SE was positively associated with extra-role performance (β = 0.342, p < 0.01). Conversely, SI negatively influenced extra-role performance (β = -0.158, p < 0.05). The model was statistically significant (F = 8.266, p < 0.001, R² = 0.215, adjusted R² = 0.195), explaining 21.5% of the variance in extra-role performance.

For overall JP, marital status and SI emerged as significant predictors (Table 6). Being married negatively influenced overall performance (β = -0.203, p < 0.01), while higher levels of SI were associated with a substantial decrease in overall JP (β = -0.450, p < 0.01). This model was statistically significant (F = 11.961, p < 0.001, R² = 0.293, adjusted R² = 0.275), explaining 29.3% of the variance in overall JP.

Table 4 presents the results of the multiple linear regression analysis examining predictors of in-role JP. The model includes marital status, SE, NA, and SI as independent variables. Unstandardized regression coefficients (β), standard errors, and p-values are displayed. Positive coefficients indicate higher in-role performance with increasing predictor values, whereas negative coefficients indicate the opposite. Statistical significance was set at p < 0.05. Model fit indices (F-value, p-value, R²) are also reported to demonstrate the explanatory power of the regression model.

**Table 4:** Summary of the multiple regression model with in-role job performance as the dependent variable. F = 8.266, p = 0.000. R² = 0.215, p < 0.05.

Variables	Β	Standard error		p
Marital status	-0.768	0.260		<0.05
Self-efficacy	0.350	0.052		<0.01
Negative affectivity	0.023	0.043		0.597
Social inhibition	-0.164	0.056		<0.05

Table 5 presents the results of the multiple linear regression analysis examining predictors of extra-role JP. Independent variables entered into the model included gender, age, overall work experience, SE, NA, and SI. Unstandardized regression coefficients (β), standard errors, and p-values are reported. Positive β values indicate an increase in extra-role performance with higher predictor levels, while negative β values indicate the opposite. Statistical significance was set at p < 0.05, and model fit indices (F value, p value, R²) are provided to illustrate the explanatory strength of the model.

**Table 5:** Summary of the multiple regression model with extra-role job performance as the dependent variable. F = 11.961, p = 0.000. R² = 0.293, p < 0.05.

Independent Variables	Β	Standard error	p
Gender	1.319	0.443	<0.05
Age	-0.015	0.068	0.825
Working experience	-0.084	0.068	0.219
Self-efficacy	0.342	0.066	<0.01
Negative affectivity	-0.066	0.058	0.260
Social inhibition	-0.158	0.072	<0.05

Table 6 presents the results of the multiple regression analysis with overall JP as the dependent variable. The model identified self-efficacy (β = 0.44, p < 0.001) and absorption, a dimension of work engagement (β = 0.48, p < 0.001), as significant positive predictors of overall performance, indicating that employees with higher self-belief and greater immersion in their work tend to perform better overall. Conversely, task delegation (β = -0.15, p = 0.003) and hindering demands limitation (β = -0.25, p = 0.008) emerged as significant negative predictors, suggesting that increased avoidance-oriented job crafting behaviors are linked to reduced performance. The overall model was statistically significant (F = 29.93, p < 0.001), explaining 46% of the variance in overall JP (R² = 0.46).

**Table 6:** Summary of the multiple regression model with overall job performance as the dependent variable. F = 7.216, p = 0.000. R² = 0.153, p < 0.05.

Independent Variables	β	Standard Error		P
Marital status	-0.203	0.564	<0.01	
Negative affectivity	0.028	0.093	0.762	
Social inhibition	-0.450	0.121	<0.01	

Multicollinearity among the independent variables for all three regressions was evaluated using the Variance Inflation Factor (VIF) values, which ranged from 1.18 to 1.79, indicating an acceptable level and no significant multicollinearity concerns. Assumptions of linear regression were further assessed through residual diagnostics. Visual examination of Q-Q plots and residual-versus-fitted value plots indicated that the residuals were approximately normally distributed and exhibited homoscedasticity, supporting the adequacy of the linear regression model.

## Discussion

The findings of the present study offer valuable insights into the psychological factors influencing JP among NGO employees. Participants reported moderate levels of NA and SI, indicating a measurable presence of Type D personality traits without extreme expression. This may indicate that NGO work, although demanding, is a calling that tends to be more psychologically resilient as well as interpersonally adaptive. On the contrary, SE scores were relatively high, indicating that employees generally considered themselves able and skilled in fulfilling their job responsibilities. This is consistent with previous findings [42,43], which underscore the positive relationship between SE and work-related outcomes. JP scores revealed that employees were performing well in both in-role and extra-role tasks, with in-role performance slightly higher on average. Importantly, SE was found to be a strong positive predictor of all JP dimensions, reinforcing the idea that belief in one’s own capabilities translates into more effective task execution and greater engagement in discretionary work behaviors. The present findings are congruent with social cognitive theory [14], which posits that individuals’ beliefs in their capability to execute specific tasks, referred to as perceived self-efficacy, exert a direct influence on their motivation, behavioral regulation, and performance outcomes. Consistent with this theoretical framework, SE emerged as a significant determinant of JP in the current study. Employees with higher levels of SE demonstrated increased confidence in their task-related abilities and exhibited elevated motivational states, which were reflected in enhanced performance across both in-role (core responsibilities) and extra-role (discretionary, organizationally beneficial) behaviors. Moreover, the present findings reinforce existing evidence, such as that by Tims et al. [44], which emphasizes the role of SE in enhancing job engagement and effectiveness. Similarly, research by Ingusci et al. [43] has shown that high SE is associated with improved in-role and extra-role performance. 

Conversely, SI emerged as a negative predictor of JP, especially for individuals with higher levels of SI, who tended to struggle with communication, collaboration, and engagement in discretionary activities, thereby limiting their overall contribution to organizational functioning. This finding is in line with previous studies, which reported that socially inhibited individuals often avoid interactions critical for team success and organizational citizenship behaviors. According to Table 3, NA showed a significant association with JP. Nevertheless, this relationship was weaker and less pervasive than reported in some prior studies. The comparatively modest effect size may reflect the moderate levels of NA in the sample or the presence of contextual buffering factors, such as meaningful work environments [45, 46]. 

Interestingly, as shown in Table 3, NA was significantly associated with JP; however, the strength of this relationship was modest across JP dimensions, which may partly diverge from prior findings in healthcare and corporate settings. One possible explanation is cultural response tendencies; for example, Greek professionals may underreport persistent negative emotions due to social desirability biases or normative emotional regulation styles [47]. Additionally, the NA subscale may not have captured domain-specific stressors relevant to NGO work, such as moral distress or mission overload, which are not always reflected in generalized affectivity measures. These contextual factors could attenuate the statistical impact of NA in multivariate models, suggesting that future studies might benefit from using more occupation-tailored affective instruments or qualitative exploration to better understand the emotional landscape of NGO personnel.

Marital status also appeared to influence JP, with married employees showing slightly lower levels of overall and in-role performance compared to their unmarried counterparts. This may be attributed to the challenges of balancing work and family responsibilities, which can lead to increased role restrain and reduced capability for sustained work engagement. This observation is consistent with Carlson et al. [48], who found that work-life conflict can undermine in-role performance. Moreover, existing literature suggests that competing demands at home may detract from employees’ availability and psychological resources in the workplace [49]. Although SE and SI were significant predictors of JP, the explained variance of the models was modest (R² = 0.153-0.293). This suggests that JP is influenced not only by individual psychological factors but also by additional organizational and contextual variables, such as organizational culture, leadership, workload, and resource availability, which should be examined in future research.

Overall, these findings underscore the importance of fostering SE and addressing the effects of SI to improve employee performance in NGOs. Organizations may benefit from targeted interventions, such as training programs to enhance SE and promote social skills, particularly for roles requiring teamwork or client interactions. Abdullah and Wider [50] have highlighted the role of SE in promoting discretionary behaviors, while Allen et al. [49] emphasize the need for work-life balance initiatives to support married employees. A recent study by Cabrera et al. [51] further demonstrates the effectiveness of structured interventions in boosting employee confidence and overall organizational contribution, making these strategies particularly relevant for the NGO context.

Strengths and limitations

This study has two main strengths. First, it contributes valuable data to the limited body of research on NGO employees, a population that is increasingly given the global expansion of NGO activities. The inclusion of personnel from diverse organizational settings enhances the contextual understanding of JB within this sector. Second, the study employed psychometrically sound instruments with high internal consistency and multiple subscales that capture a wide range of psychological and performance -related constructs.

However, several limitations must be acknowledged. The sample was relatively small, and the participants’ heterogeneity limits the generalizability of the findings to the broader NGO workforce. Moreover, data were obtained using a self-reported measure, and participants may have answered the questionnaire in a socially desirable manner so they would not expose themselves. Also, the study employed convenience sampling, and the response rate could not be determined, as only completed questionnaires were accessible to the research team. This may introduce selection bias and limit the generalizability of the findings. Finally, the study’s design is cross-sectional, which means that it studies a random sample in a specific geographical space and time. As a result, it does not take into account the sequence of causality between dependent variables and JP. Future longitudinal or experimental research is needed to establish directional relationships and to validate the present findings across diverse NGO environments.

Implications and future directions

Beyond their theoretical contribution, the present findings carry important practical implications for NGO contexts. Interventions that strengthen SE and reduce the behavioral impact of SI may support both in-role and extra-role performance. At the same time, the modest explanatory power of the models suggests that future research should examine additional organizational and contextual determinants of JP, ideally through longitudinal and mixed-method designs.

## Conclusions

This study underscores the critical role of psychological characteristics, namely, SE and SI, in shaping JP among employees in the NGO sector. The results confirm that higher SE is consistently associated with stronger in-role and extra-role performance, while elevated SI negatively impacts both domains. These findings provide a valuable psychological lens through which the dynamics of performance in nonprofit organizations can be better understood and strategically supported. Future studies are encouraged to explore additional organizational variables and employ longitudinal methodologies to clarify causal pathways and enhance generalizability.

In light of these outcomes, NGOs, especially within Greece’s socioeconomically constrained environment, should prioritize organizational strategies that cultivate SE while mitigating the impact of SI. Practical interventions might include structured training programs in cognitive-behavioral approaches, strengths-based mentorship systems, and role-tailored job crafting initiatives aimed at reinforcing employees’ confidence and perceived competence. Simultaneously, fostering psychologically safe workspaces, characterized by trust, inclusive communication, and openness to feedback, could reduce the inhibitory effects of SI, particularly in roles requiring interpersonal engagement and emotional labor. From a policy perspective, both national-level NGO frameworks and internal governance bodies should consider embedding employee psychological resilience into broader human resources and quality assurance structures. Initiatives supporting work-life balance, continuous professional development, and routine well-being monitoring could yield significant returns not only in performance outcomes but also in employee retention and organizational sustainability. These strategies are particularly critical for Greek NGOs, where persistent financial constraints and elevated levels of workforce burnout have long undermined organizational continuity and human capital development.

Future research should include longitudinal designs to explore causal mechanisms and trajectories of SE and Type D personality traits over time. Additionally, qualitative investigations could help contextualize the lived experiences behind high or low performance in NGO settings, especially in relation to emotional demands and value-driven motivation. Cross-cultural comparisons may also reveal how cultural norms regarding emotional expression and self-perception influence the expression and impact of SI and SE across nonprofit environments. Ultimately, a deeper understanding of the psychological architecture underpinning JP in NGOs can guide more effective human capital development strategies, allowing organizations to fulfill their missions with greater stability, adaptability, and impact.
